# Stability analysis of nonlinear algebraic-differential equations with 2-delays and numerical methods

**DOI:** 10.1371/journal.pone.0347322

**Published:** 2026-04-21

**Authors:** Huiqing Liao, Qi Li, Xunpeng Xia, Dongjie Tang

**Affiliations:** 1 Department of Customs Inspection and Quarantine, Shanghai Customs University, Shanghai, China; 2 Fundamental Teaching Department, Shanghai Customs University, Shanghai, China; Khalifa University of Science and Technology, UNITED ARAB EMIRATES

## Abstract

This paper investigates stability analysis and numerical approaches for nonlinear delay differential-algebraic equations with 2-delays. Based on asymptotic stability considerations for Hessenberg type delay differential-algebraic equations, we develop a stability analysis approach using direct linearization. This analysis provides some insight into how stability properties can be preserved under discretization. The main contribution of this paper lies in establishing a stability analysis system based on local linearization, and consists in deriving sufficient conditions for the implicit Euler method and the 2-step backward differentiation formula method to preserve stability and asymptotic stability properties of the nonlinear continuous system. Numerical results demonstrate that the proposed numerical methods can effectively maintain the stability of the original systems, thereby supporting the practical solution of delay differential-algebraic equations.

## 1 Introduction

Time-delayed systems of differential equations arise naturally in mathematical modeling wherever the present behavior of a system is influenced by its prior state, when the dynamics possess an inherent dependence on past history [1–5]. Meanwhile, differential-algebraic equations (DAEs) are employed to model chemical engineering, epidemiology and ecology processes, such as flash separation, where a framework based on DAEs describes how a feed stream separates into liquid and vapor phases under given thermodynamic conditions [6–9]. Systems incorporating time delays alongside algebraic constraints are described as time delay differential-algebraic equations (DDAEs), this class of equations finds significant application in engineering [10–12]. During a chemical reaction, the concentration change of the reactant is often affected by the concentration at the past moment. With the development of mathematical models, neural networks, computer simulation, and artificial intelligence, DDAEs describe this chemical reaction process with time delay, thus helping chemists to conduct more accurate reaction simulation and optimization.

It is known that the implicit equations, even in the case of an algebraic equation, are very complicated and difficult for mathematical numerical treatment [13]. This is exemplified by studies of complex dynamical systems governed by nonlinear equations, such as the nonlinear motion of a double pendulum [14] or the rotational motion of a rigid body under external and gyroscopic torques [15]. Given the impracticality of obtaining exact solutions for such systems, a prevalent approach is to employ asymptotic methods (e.g., the multiple scales technique) to derive approximate analytical solutions, and to complement these with numerical simulations to analyze stability and dynamical behavior [14–18]. Since directly solving most DAEs and DDAEs for exact solutions is highly challenging, therefore, researchers focus on analyzing the stability and asymptotic stability of numerical solutions [19–28].

The numerical analysis of DAEs and DDAEs has primarily focused on linear systems, where significant progress has been achieved. Various numerical methods have been developed and analyzed for linear DAEs and DDAEs, including θ-methods, Runge-Kutta (R-K) methods, and linear multistep methods [19,22,24–27,29–32]. These approaches have successfully addressed key numerical issues such as stability, asymptotic stability preservation, order reduction phenomena, and consistent initialization procedures for linear systems. However, the extension of these methods to nonlinear DDAEs presents considerably challenges and remains substantially less explored in literatures. The primary difficulties stem from the intricate interplay between the inherent stiffness of DAEs, the non-local nature of delay terms, and the nonlinear coupling between solution components. These compounded challenges have limited the development of general-purpose numerical schemes for nonlinear DDAEs, with most existing studies restricted to specific sub-classes or requiring restrictive assumptions on system structure [20,33].

This paper presents a novel approach to the analysis of numerical methods for nonlinear DDAEs with 2-delays, building upon the asymptotic stability theory for Hessenberg DDAEs established in [34]. The primary contribution is a stability analysis framework based on direct linearization, which allows for the derivation of sufficient conditions under which numerical discretizations, such as the implicit Euler method and the 2-step backward differentiation formula (BDF) method, and preserve the stability and asymptotic stability properties of the original nonlinear DDAEs. In particular, we examine the extent to which stability characteristics are preserved when transitioning from the continuous linearized system to its discrete counterpart. This study provides rigorously justified stability criteria that support the reliable application of practical computational methods.

The paper is organized as follows. In the next section, we present a direct linearization method for nonlinear DDAEs with 2-delays. The main contributions of this work are presented in Section 3, where sufficient conditions are established to ensure the stability of nonlinear DDAEs with 2-delays. Additionally, several practical criteria for the asymptotic stability of such systems are proposed. In Section 4, the stability of numerical solutions is analyzed using the implicit Euler method and the 2-step BDF method. Numerical experiments are provided in Section 5. Finally, the concluding remarks and future perspectives on numerical methods for nonlinear DDAEs with multiple delays are discussed in the last section.

## 2 Direct linearization method for nonlinear DDAEs

Let ${\mathbb{R}}^{d}$ be a *d*-dimensional Euclidian space with the inner product <·,·> and the norm ‖·‖ induced by inner product. We consider the initial value problem (IVPs) of nonlinear DDAEs with 2-delays of the general form


$$\begin{array}{c} \left\{ \begin{array}{cc} u^{\prime }(t)=f\left(t,u(t),u(t-\tau ),v(t),v(t-\tau )\right),\; & t\in (0,T],\\ 0=g(t,u(t),u(t-\tau ),v(t)),\; & t\in (0,T],\\ u(t)=\Phi (t),\;v(t)=\Psi (t),\; & t\in [-2\tau ,0], \end{array}\right. \end{array}$$ (1)

where


$$\Phi (t)=\left\{ \begin{array}{c} \phi_{1}(t),\;t\in [-\tau ,0],\\ \phi_{2}(t),\;t\in [-2\tau ,-\tau ), \end{array}\right.\;\Psi (t)=\left\{ \begin{array}{c} \psi_{1}(t),\;t\in [-\tau ,0],\\ \psi_{2}(t),\;t\in [-2\tau ,-\tau ), \end{array}\right.$$

and τ, *T* are positive constants, 0≤τ≤∞, 0 < *T*≤ ∞.

As proven in [30,34], directly linearizing a DDAE yields a system equivalent to linearizing its state space form, both producing identical linear delay ordinary differential equations. This equivalence, derived from the implicit function theorem and stationary solutions, validates direct linearization as mathematically sound. Assuming that $\frac{\partial g}{\partial v}$ is nonsingular, hence, the implicit function theorem enables us to solve the DDAEs with 2-delays (1) for *v*(*t*), which is expressed as


v(t)=ρ(t,u(t),u(t−τ)),  t>0,  τ>0.

Substituting the above inequation into (1) yields the delay ordinary differential equations (DODEs)


$$u^{\prime }(t)=f\left(t,u(t),u(t-\tau ),\rho \left(t,u(t),u(t-\tau ),u(t-2\tau )\right)\right).$$

Consequently, the nonlinear DDAEs with 2-delays (1) are stable if and only if the DODEs is stable. If all delayed terms are to be explicitly represented in this DODEs, the initial data must be defined on the interval t∈[−2τ,0]. To simplify the notation in the following sections, u(t−τ), u(t−2τ), and v(t−τ) are abbreviated as $u_{\tau }$, $u_{2\tau }$, and $v_{\tau }$, respectively. In the following analysis, a linearization approach is developed for nonlinear DDAEs with 2-delays (1). Our analysis is restricted to cases where a stationary solution exists and is valid only within a local neighborhood of that solution. Under the assumption of local linearizability, we develop a new stability criterion for such nonlinear systems, which is simpler to apply than the one given in [33]. The method involves linearizing the nonlinear DDAEs, applying the implicit function theorem, utilizing vector inner products and consistent initial vectors, and finally establishing stability and asymptotic stability conditions.

We assume that $\frac{\partial f}{\partial u}$, $\frac{\partial f}{\partial v}$, $\frac{\partial f}{\partial u_{\tau }}$, $\frac{\partial f}{\partial v_{\tau }}$, $\frac{\partial g}{\partial u}$, $\frac{\partial g}{\partial v}$, and $\frac{\partial g}{\partial v_{\tau }}$ are coefficients that depend only on $u,v,u_{\tau },v_{\tau }$ and remain bounded in a neighborhood of the stationary solution. Furthermore, ${\left(\frac{\partial g}{\partial v}\right)}^{-1}$ and ${\left(\frac{\partial g}{\partial v_{\tau }}\right)}^{-1}$ are also assumed to exist and be bounded. Direct linearization of original problem (1) at the neighborhood of stationary solution yields


$$\begin{array}{c} \left\{ \begin{array}{cc} u^{\prime }=\frac{\partial f}{\partial u}u+\frac{\partial f}{\partial u_{\tau }}u_{\tau }+\frac{\partial f}{\partial v}v+\frac{\partial f}{\partial v_{\tau }}v_{\tau },\; & t\in (0,T],\\ 0=\frac{\partial g}{\partial u}u+\frac{\partial g}{\partial u_{\tau }}u_{\tau }+\frac{\partial g}{\partial v}v,\; & t\in (0,T],\\ u(t)=\Phi (t),\;v(t)=\Psi (t),\; & t\in [-2\tau ,0], \end{array}\right. \end{array}$$ (2)

where


$$\Phi (t)=\left\{ \begin{array}{c} \phi_{1}(t),\;t\in [-\tau ,0],\\ \phi_{2}(t),\;t\in [-2\tau ,-\tau ), \end{array}\right.\;\Psi (t)=\left\{ \begin{array}{c} \psi_{1}(t),\;t\in [-\tau ,0],\\ \psi_{2}(t),\;t\in [-2\tau ,-\tau ). \end{array}\right.$$

According to linear analysis only applied locally to consider trends of small perturbation at the neighborhood of stationary solution [19], we ignore the higher order term $r(t,u,\tilde{u},v,\tilde{v},u_{\tau },{\tilde{u}}_{\tau },v_{\tau },{\tilde{v}}_{\tau })$ in Taylor expansion. The perturbation of system (2) yields the following equations


$$\begin{array}{c} \left\{ \begin{array}{cc} {\tilde{u}}^{\prime }=\frac{\partial f}{\partial u}\tilde{u}+\frac{\partial f}{\partial u_{\tau }}{\tilde{u}}_{\tau }+\frac{\partial f}{\partial v}\tilde{v}+\frac{\partial f}{\partial v_{\tau }}{\tilde{v}}_{\tau },\; & t\in (0,T],\\ 0=\frac{\partial g}{\partial u}\tilde{u}+\frac{\partial g}{\partial u_{\tau }}{\tilde{u}}_{\tau }+\frac{\partial g}{\partial v}\tilde{v},\; & t\in (0,T],\\ \tilde{v}(t)=\tilde{\Psi }(t),\;\tilde{v}(t)=\tilde{\Psi }(t),\; & t\in [-2\tau ,0], \end{array}\right. \end{array}$$ (3)

where


$$\begin{array}{c} \tilde{\Phi }(t)=\left\{ \begin{array}{cc} {\tilde{\phi }}_{1}(t),\; & t\in [-\tau ,0],\\ {\tilde{\phi }}_{2}(t),\; & t\in [-2\tau ,\tau ), \end{array}\right.\;\tilde{\Psi }(t)=\left\{ \begin{array}{cc} {\tilde{\psi }}_{1}(t),\; & t\in [-\tau ,0],\\ {\tilde{\psi }}_{2}(t),\; & t\in [-2\tau ,\tau ). \end{array}\right. \end{array}$$

To analyze the stability of DDAEs with 2-delays (2), we recall the standard definitions of stability and asymptotic stability in the context of DDAEs [33,35].

**Definition 2.1** *[35] The nonlinear DDAEs* (1) *are stable, if inequalities*


$$\begin{array}{cc} \left\|u(t)-\tilde{u}(t)\right\| & \leq \max_{-2\tau \leq t\leq 0}\|\Phi (t)-\tilde{\Phi }(t)\|,\\ \left\|v(t)-\tilde{v}(t)\right\| & \leq L\max_{-2\tau \leq t\leq 0}\|\Phi (t)-\tilde{\Phi }(t)\|, \end{array}$$

*are satisfied, where L > 0 is a constant.*

**Definition 2.2** *[33] The nonlinear DDAEs are asymptotically stable if and only if for every consistent initial value function* Φ(t)*,* Ψ(t)*,* $\tilde{\Phi }(t)$*,* $\tilde{\Psi }(t)$*. and solutions {u(*t*),v(*t*)},* $\left\{\tilde{u}(t),\tilde{v}(t)\right\}$ *satisfy*


$$\lim_{t\to \infty }\|u(t)-\tilde{u}(t)\|=0,\;\lim_{t\to \infty }\|v(t)-\tilde{v}(t)\|=0.$$

## 3 Stability results for analytic solution of nonlinear DDAEs with 2-delays

To study the stability and asymptotic stability of DDAEs, it is necessary to introduce following two Lemmas [35,36].

**Lemma 3.1.** *[35,36] Consider the following IVPs*


$$\chi^{\prime }(t)=a(t)\chi (t)+\eta (t),\;\chi (0)=\chi_{0},\;t>0,$$ (4)

*where a(t),* χ(t) *are continuous function of t when* *t* ≥ 0*, Re(a(t))<0. Then, the solution of IVPs (4) satisfies*


$$|\chi (t)|\leq \max\left\{|\chi (0)|,\;\max_{0\leq s\leq t}\frac{\left|\eta (s)\right|}{-Re(a(s))}\right\},\;t\geq 0.$$

**Lemma 3.2.** *[35,36] Suppose a non-negative function Z(t) satisfies*


$$\begin{array}{c} \left\{ \begin{array}{cc} Z^{\prime }(t)=\omega (t)Z(t)+\gamma_{1}(t)Z(t-\tau )+\gamma_{2}(t)Z(t-2\tau ),\; & t>0,\;\tau >0,\\ Z(t)=\varphi (t),\; & t\leq 0. \end{array}\right. \end{array}$$

*where* $\varphi (t)\geq 0,\;\omega (t),\;\gamma_{1}(t)$*, and* $\gamma_{2}(t)$ *are given functions and*


$$\begin{array}{cc} \gamma_{1}(t)+\gamma_{2}(t)\leq -\xi \omega (t),\; & 0\leq \xi <1,\;\forall t\geq 0\\ \omega (t)\leq -\beta <0,\; & \forall t\geq 0. \end{array}$$

*Then,* Z(t)→0(t→0).

These two Lemmas serve as fundamental comparison tools for our stability and asymptotic stability analysis of DDAEs. Lemma 3.1 provides an a priori bound for solutions of linear non-homogeneous systems, which will be used to control the perturbation terms arising from linearization. Lemma 3.2 offers a decay criterion for scalar delay differential inequalities, upon applying appropriate inner product operations, this criterion can be applied to the norm of the solution, thereby enabling a systematic treatment of the delay terms.

### 3.1 Stability analysis

For a symmetric negative matrix *P*(*t*), by the Schur Decomposition Theorem [37], we find an orthogonal matrix *H*, such that


$$H^{T}P(t)H=diag\left(\lambda_{1},\lambda_{2},\lambda_{3}\cdots ,\lambda_{n}\right),\;\lambda_{i}<0,\;(i=1,2,3\cdots n).$$ (5)

Assuming initial values satisfy the existence of $(\frac{\partial g}{\partial v_{\tau }}{)}^{-1}$, and that $g(u_{\tau },u_{2\tau },v_{\tau })=0$, we set


$$x=H^{T}u,\;y=H^{T}v,\;x_{\tau }=H^{T}u_{\tau },\;y_{\tau }=H^{T}v_{\tau }.$$ (6)

Then, multiplying both sides of the first two equations in systems (2) and (3) yields


$$\left\{ \begin{array}{c} x^{\prime }=H^{T}(\frac{\partial f}{\partial u}H\cdot x+\frac{\partial f}{\partial u_{\tau }}H\cdot x_{\tau }+\frac{\partial f}{\partial v}H\cdot y+\frac{\partial f}{\partial v_{\tau }}H\cdot y_{\tau }),\\ 0=H^{T}(\frac{\partial g}{\partial u}H\cdot x+\frac{\partial g}{\partial u_{\tau }}H\cdot x_{\tau }+\frac{\partial g}{\partial v}H\cdot y), \end{array}\right.$$ (7)

and


$$\left\{ \begin{array}{c} {\tilde{x}}^{\prime }=H^{T}(\frac{\partial f}{\partial u}H\cdot \tilde{x}+\frac{\partial f}{\partial u_{\tau }}H\cdot {\tilde{x}}_{\tau }+\frac{\partial f}{\partial v}H\cdot \tilde{y}+\frac{\partial f}{\partial v_{\tau }}H\cdot {\tilde{y}}_{\tau }),\\ 0=H^{T}(\frac{\partial g}{\partial u}H\cdot \tilde{x}+\frac{\partial g}{\partial u_{\tau }}H\cdot {\tilde{x}}_{\tau }+\frac{\partial g}{\partial y}H\cdot \tilde{y}), \end{array}\right.$$ (8)

where *t* and τ are positive constants.

From the second equation in (7) and (8), we obtain


$$y=-H^{T}(\frac{\partial g}{\partial v}{)}^{-1}\frac{\partial g}{\partial u}H\cdot x-H^{T}(\frac{\partial g}{\partial v}{)}^{-1}\frac{\partial g}{\partial u_{\tau }}H\cdot x_{\tau },$$

and


$$\tilde{y}=-H^{T}(\frac{\partial g}{\partial v}{)}^{-1}\frac{\partial g}{\partial u}H\cdot \tilde{x}-H^{T}(\frac{\partial g}{\partial v}{)}^{-1}\frac{\partial g}{\partial u_{\tau }}H\cdot {\tilde{x}}_{\tau }.$$

Taking the difference of the two equations above and making use of (7) and (8) yields


$$\begin{array}{c} y-\tilde{y}=-H^{T}(\frac{\partial g}{\partial v}{)}^{-1}\frac{\partial g}{\partial u}H\cdot (x-\tilde{x})-H^{T}(\frac{\partial g}{\partial v}{)}^{-1}\frac{\partial g}{\partial u_{\tau }}H\cdot (x_{\tau }-{\tilde{x}}_{\tau }),\\ y_{\tau }-{\tilde{y}}_{\tau }=-H(\frac{\partial g}{\partial v_{\tau }}{)}^{-1}\frac{\partial g}{\partial u_{\tau }}H\cdot (x_{\tau }-{\tilde{x}}_{\tau })-H(\frac{\partial g}{\partial v_{\tau }}{)}^{-1}\frac{\partial g}{\partial u_{2\tau }}H\cdot (x_{2\tau }-{\tilde{x}}_{2\tau }). \end{array}$$ (9)

The specific proof process of the stability theorem will be presented in the following.

**Theorem 3.1.** *Suppose t is in a domain of some neighborhood of a stationary solution, and let*


$$\begin{array}{cc} & P(t)=\frac{\partial f}{\partial u}-\frac{\partial f}{\partial v}(\frac{\partial g}{\partial v}{)}^{-1}\frac{\partial g}{\partial u},\;R(t)=\frac{\partial f}{\partial v_{\tau }}(\frac{\partial g}{\partial v_{\tau }}{)}^{-1}\frac{\partial g}{\partial u_{2\tau }},\\ & Q(t)=\frac{\partial f}{\partial u_{\tau }}-\frac{\partial f}{\partial v}(\frac{\partial g}{\partial v}{)}^{-1}\frac{\partial g}{\partial u_{\tau }}-\frac{\partial f}{\partial v_{\tau }}(\frac{\partial g}{\partial v_{\tau }}{)}^{-1}\frac{\partial g}{\partial u_{\tau }}. \end{array}$$

*P(t) is a symmetric matrix and its eigenvalues satisfy*


$$\lambda_{n}(t)\leq \lambda_{n-1}(t)\leq \cdots \leq \lambda_{1}(t)<0.$$

*Let* γ(t)=‖Q(t)‖, ε(t)=‖R(t)‖*, if*


$$\gamma (t)+\varepsilon (t)<-\lambda_{1}(t),$$ (10)

equations (1) *are stable.*

**Proof.** *Letting*


$$\sigma (t)=x-\tilde{x}=H^{T}(u-\tilde{u}),\;\sigma (t-\tau )=x_{\tau }-{\tilde{x}}_{\tau }=H^{T}(u_{\tau }-{\tilde{u}}_{\tau }),$$

*and subtracting* equations (7) *and* (8)*, we obtain from (9) that*


$$\begin{array}{c} \begin{array}{cc} \sigma (t) & =H^{T}(\frac{\partial f}{\partial u}-\frac{\partial f}{\partial v}(\frac{\partial g}{\partial v}{)}^{-1}\frac{\partial g}{\partial u})H\cdot (x-\tilde{x})\\ & +H^{T}(\frac{\partial f}{\partial v_{\tau }}(\frac{\partial g}{\partial v_{\tau }}{)}^{-1}\frac{\partial g}{\partial u_{2\tau }})H\cdot (x_{2\tau }-{\tilde{x}}_{2\tau })\\ & +H^{T}(\frac{\partial f}{\partial u_{\tau }}-\frac{\partial f}{\partial v}(\frac{\partial g}{\partial v}{)}^{-1}\frac{\partial g}{\partial u_{\tau }}-\frac{\partial f}{\partial v_{\tau }}(\frac{\partial g}{\partial v_{\tau }}{)}^{-1}\frac{\partial g}{\partial u_{\tau }})H\cdot (x_{\tau }-{\tilde{x}}_{\tau })\\ & =H^{T}P(t)H\cdot \sigma (t)+H^{T}Q(t)H\cdot \sigma (t-\tau )+H^{T}R(t)H\cdot \sigma (t-2\tau ). \end{array} \end{array}$$ (11)

*Letting* Y(t)=‖σ(t)‖ *and taking inner product of* $\sigma^{\prime }(t)$ *and* σ(t) *to get*


$$\begin{array}{c} \begin{array}{cc} & \;Y^{\prime }(t)\cdot Y(t)=<\sigma^{\prime }(t),\sigma (t)>\\ & =<H^{T}P(t)H\cdot \sigma (t)+H^{T}Q(t)H\cdot \sigma (t-\tau )+H^{T}R(t)H\cdot \sigma (t-2\tau ),\sigma (t)>\\ & \leq \sigma (t{)}^{T}\cdot H^{T}P(t)H\cdot \sigma (t)+\|Q(t)\|\cdot Y(t-\tau )\cdot Y(t)\\ & +\|R(t)\|\cdot Y(t-2\tau )\cdot Y(t)\\ & \leq \lambda (t)\cdot Y(t{)}^{2}+\gamma (t)\cdot Y(t-\tau )\cdot Y(t)+\varepsilon (t)\cdot Y(t-2\tau )\cdot Y(t). \end{array} \end{array}$$ (12)

*Due to* *Y*(*t*) ≠ 0*, we divide both sides of the inequality (12) by Y(t) to get*


$$Y^{\prime }(t)\leq \lambda (t)\cdot Y(t)+\gamma (t)\cdot Y(t-\tau )+\varepsilon (t)\cdot Y(t-2\tau ).$$ (13)

*Considering the following IVPs for delay differential equations (DDEs)*


$$\begin{array}{c} \left\{ \begin{array}{cc} {\tilde{Y}}^{\prime }(t) & =\lambda (t)\cdot \tilde{Y}(t)+\gamma (t)\cdot \tilde{Y}(t-\tau )+\varepsilon (t)\cdot \tilde{Y}(t-2\tau ),\\ \tilde{Y}(0) & =\left\|H^{T}\cdot (\Phi (0)-\tilde{\Phi }(0))\right\|. \end{array}\right. \end{array}$$ (14)

*Using the equations above, condition (10), and Lemma 3.1, we obtain*


$$\begin{array}{c} \begin{array}{cc} \tilde{Y}(t) & \leq \max\left\{\left\|H^{T}\cdot (\Phi (0)-\tilde{\Phi }(0))\right\|,\;\max_{0\leq s\leq t}\frac{\gamma \cdot \|\tilde{Y}(s-\tau )\|+\varepsilon \cdot \|\tilde{Y}(s-2\tau )\|}{-\lambda }\right\}\\ & \leq \max\left\{\|H^{T}\|\cdot \|\Phi (0)-\tilde{\Phi }(0)\|,\;\max_{0\leq s\leq t}Y(s-\tau ),\;\max_{0\leq s\leq t}Y(s-2\tau )\right\}\\ & \leq \max_{-2\tau \leq t\leq 0}\|\Phi (t)-\tilde{\Phi }(t)\|. \end{array} \end{array}$$

*Due to*


$$\|Y(t)\|=\|H^{T}\|\cdot \|u(t)-\tilde{u}(t)\|,\;\|H^{T}\|\neq 0,\;\|Y(t)\|<\|\tilde{Y}(t)\|,$$ (15)

*we apply mathematics induction to have*


$$\|u(t)-\tilde{u}(t)\|\leq \max_{-2\tau \leq t\leq 0}\|\Phi (t)-\tilde{\Phi }(t)\|,\;\forall t\geq 0.$$

*Similar to* $\left\|u(t)-\tilde{u}(t)\right\|$*, in the case of* $\left\|v(t)-\tilde{v}(t)\right\|$*, it follows from (9) and*


$$\|y(t)-\tilde{y}(t)\|=\|H^{T}\|\cdot \|v(t)-\tilde{v}(t)\|,$$

*we obtain*


$$\begin{array}{c} \begin{array}{cc} & \;\|v(t)-\tilde{v}(t)\|\\ & \leq \underset{u,v,u_{\tau }\in R^{n}}{\sup}\left\{\left\|{\left(\frac{\partial g}{\partial v}\right)}^{-1}\frac{\partial g}{\partial u}\right\|+\left\|{\left(\frac{\partial g}{\partial v}\right)}^{-1}\frac{\partial g}{\partial u_{\tau }}\right\|\right\}\cdot \max_{-2\tau \leq t\leq 0}\|\Phi (t)-\tilde{\Phi }(t)\|\\ & =L\max_{-2\tau \leq t\leq 0}\|\Phi (t)-\tilde{\Phi }(t)\|, \end{array} \end{array}$$

*where*


$$L=\underset{u,v,u_{\tau }\in R^{n}}{\sup}\left\{\left\|{\left(\frac{\partial g}{\partial v}\right)}^{-1}\frac{\partial g}{\partial u}\right\|+\left\|{\left(\frac{\partial g}{\partial v}\right)}^{-1}\frac{\partial g}{\partial u_{\tau }}\right\|\right\}.$$ (16)

### 3.2 Asymptotic stability

**Theorem 3.2.** *Under the conditions stated above Theorem 3.1, if systems (2) satisfies*


$$0<-\lambda_{1}(t)\leq \beta (t),\;\gamma (t)+\varepsilon (t)\leq -\xi \lambda_{1}(t),\;0\leq \xi <1.$$

*Then,* equations (1) *are asymptotically stable.*

**Proof.** *Similarly, we consider the IVPs of DDEs* (14), *under the conditions of Lemma 3.2, we obtain*


$$\lim_{t\to \infty }\|\tilde{Y}(t)\|=0.$$

*Since the inequality* $\left\|Y(t)\|<\|\tilde{Y}(t)\right\|$ *holds, it follows that*


$$\lim_{t\to \infty }\|Y(t)\|=0.$$

*From (15) and*


$$\|y(t)-\tilde{y}(t)\|=\|H^{T}\|\cdot \|v(t)-\tilde{v}(t)\|,$$

*it is easy to verify for* $\left\|u(t)-\tilde{u}(t)\right\|$ *and* $\left\|v(t)-\tilde{v}(t)\right\|$ *that*


$$\lim_{t\to \infty }\|u(t)-\tilde{u}(t)\|=0,\;\lim_{t\to \infty }\|v(t)-\tilde{v}(t)\|\leq L\lim_{t\to \infty }\|u(t)-\tilde{u}(t)\|=0,$$

*where L is expressed as (16).*

## 4 Stability and asymptotic stability applying numerical methods for nonlinear DDAEs with 2-delays

Let *u*_*n*_, *v*_*n*_, ${\tilde{u}}_{n}$, and ${\tilde{v}}_{n}$ denote the numerical solutions of (2) and (3), respectively. Following Definitions 2.1 and 2.2, we now introduce the definitions of stability and asymptotic stability for the numerical solutions of DDAEs [33].

**Definition 4.1** *[33] A numerical method for solving DDAEs is called stable, if for every consistent initial value functions* Φ(t)*,* Ψ(t)*,* $\tilde{\Phi }(t)$*,* $\tilde{\Psi }(t)$*, and each step h > 0, the solution sequences* {*u*_*n*_, *v*_*n*_}*,* $\left\{{\tilde{u}}_{n},{\tilde{v}}_{n}\right\}$ *satisfy*


$$\begin{array}{c} \begin{array}{cc} \|u_{n}-{\tilde{u}}_{n}\|\leq \max_{-2\tau \leq t\leq 0}\|\Phi (t)-\tilde{\Phi }(t)\|,\; & n=1,2,\cdots ,\\ \|v_{n}-{\tilde{v}}_{n}\|\leq L\max_{-2\tau \leq t\leq 0}\|\Phi (t)-\tilde{\Phi }(t)\|,\; & n=1,2,\cdots . \end{array} \end{array}$$

**Definition 4.2** *[33] A numerical method for solving DDAEs is called asymptotically stable, if for every consistent initial value function* Φ(t)*,* Ψ(t)*,* $\tilde{\Phi }(t)$*,* $\tilde{\Psi }(t)$*, and each step h > 0, the solution sequences* {*u*_*n*_, *v*_*n*_}*,* $\left\{{\tilde{u}}_{n},{\tilde{v}}_{n}\right\}$ *satisfy*


$$\lim_{n\to \infty }\|u_{n}-{\tilde{u}}_{n}\|=0,\;\lim_{n\to \infty }\|v_{n}-{\tilde{v}}_{n}\|=0,\;n=1,2,\cdots .$$

### 4.1 The implicit Euler method

Let $u_{n}=u_{n}(h),\;v_{n}=v_{n}(h)$, where *h* > 0 denotes the step size. Applying the implicit Euler method to (1) yields


$$\begin{array}{c} \left\{ \begin{array}{cc} u_{n+1} & =u_{n}+f(t_{n+1},u_{n+1},u_{n+1-m},v_{n+1},v_{n+1-m}),\;n=1,2,\cdots \\ 0 & =g(u_{n+1},u_{n+1-m},v_{n+1}),\\ u_{n} & =\Phi_{1}(t_{n}),\;v_{n}=\Psi (t_{n}),\;-m\leq n\leq 0,\;(mh=\tau ,\;m\geq 1), \end{array}\right. \end{array}$$ (17)

where


$$\begin{array}{c} u_{n}=\Phi (t_{n})=\left\{ \begin{array}{cc} \phi_{1}(t_{n}),\; & t_{n}\in [-\tau ,0],\\ \phi_{2}(t_{n}),\; & t_{n}\in [-2\tau ,\tau ), \end{array}\right. \end{array}$$

$$\begin{array}{c} v_{n}=\Psi (t_{n})=\left\{ \begin{array}{cc} \psi_{1}(t_{n}),\; & t_{n}\in [-\tau ,0],\\ \psi_{2}(t_{n}),\; & t_{n}\in [-2\tau ,\tau ), \end{array}\right. \end{array}$$

Linearizing equations (17) directly in a neighborhood of the stationary solution gives


$$\begin{array}{c} \left\{ \begin{array}{cc} u_{n+1} & =u_{n}+h\left(\frac{\partial f}{\partial u_{n+1}}u_{n+1}+\frac{\partial f}{\partial u_{n+1-m}}u_{n+1-m}\right)\\ & +h\left(\frac{\partial f}{\partial v_{n+1}}v_{n+1}+\frac{\partial f}{\partial v_{n+1-m}}v_{n+1-m}\right),\;n=1,2,\cdots ,\\ 0 & =\frac{\partial g}{\partial u_{n+1}}u_{n+1}+\frac{\partial g}{\partial u_{n+1-m}}u_{n+1-m}+\frac{\partial g}{\partial v_{n+1}}v_{n+1},\\ u_{n} & =\Phi (t_{n}),\;v_{n}=\Psi (t_{n}),\;-m\leq n\leq 0,\;(mh=\tau ,\;m\geq 1). \end{array}\right. \end{array}$$ (18)

The perturbed form of [18] are


$$\begin{array}{c} \left\{ \begin{array}{cc} {\tilde{u}}_{n+1} & ={\tilde{u}}_{n}+h\left(\frac{\partial f}{\partial u_{n+1}}{\tilde{u}}_{n+1}+\frac{\partial f}{\partial u_{n+1-m}}{\tilde{u}}_{n+1-m}\right)\\ & +h\left(\frac{\partial f}{\partial v_{n+1}}{\tilde{v}}_{n+1}+\frac{\partial f}{\partial v_{n+1-m}}{\tilde{v}}_{n+1-m}\right),\;n=1,2,\cdots ,\\ & 0=\frac{\partial g}{\partial u_{n+1}}{\tilde{u}}_{n+1}+\frac{\partial g}{\partial u_{n+1-m}}{\tilde{u}}_{n+1-m}+\frac{\partial g}{\partial v_{n+1}}{\tilde{v}}_{n+1},\\ {\tilde{u}}_{n} & =\tilde{\Phi }(t_{n}),\;{\tilde{v}}_{n}=\tilde{\Psi }(t_{n}),\;-m\leq n\leq 0,\;(mh=\tau ,\;m\geq 1), \end{array}\right. \end{array}$$ (19)

where


$$\begin{array}{c} {\tilde{u}}_{n}=\tilde{\Phi }(t_{n})=\left\{ \begin{array}{cc} {\tilde{\phi }}_{1}(t_{n}),\; & t_{n}\in [-\tau ,0],\\ {\tilde{\phi }}_{2}(t_{n}),\; & t_{n}\in [-2\tau ,\tau ), \end{array}\right. \end{array}$$

$$\begin{array}{c} {\tilde{v}}_{n}=\tilde{\Psi }(t_{n})=\left\{ \begin{array}{cc} {\tilde{\psi }}_{1}(t_{n}),\; & t_{n}\in [-\tau ,0],\\ {\tilde{\psi }}_{2}(t_{n}),\; & t_{n}\in [-2\tau ,\tau ). \end{array}\right. \end{array}$$

Similar to the stability analysis in 3.1, there exists an orthogonal matrix *H* satisfies (5). Since *H*^*T*^*P*(*t*)*H* is a negative definite diagonal matrix, it follows that


$$\left\|{\left(I-hH^{T}P(t)H\right)}^{-1}\right\|\leq \frac{1}{1-h\lambda_{1}(t)}.$$ (20)

Following a procedure analogous to that outlined in equations (6)-(9), and letting


$$\sigma_{n}=H^{T}\cdot (u_{n}-{\tilde{u}}_{n}),\;\sigma_{n+1}=H^{T}\cdot (u_{n+1}-{\tilde{u}}_{n+1}),\;\sigma_{n+1-m}=H^{T}\cdot (u_{n+1-m}-{\tilde{u}}_{n+1-m}),$$

we combine (18) and (19) to obtain a result similar to (11) as follows


$$\sigma_{n+1}=\sigma_{n}+h\left(H^{T}P(t)H\cdot \sigma_{n+1}+H^{T}Q(t)H\cdot \sigma_{n+1-m}+H^{T}R(t)H\cdot \sigma_{n+1-2m})\right),$$

where


$$\begin{array}{c} \begin{array}{cc} P(t) & =\frac{\partial f}{\partial u_{n+1}}-\frac{\partial f}{\partial v_{n+1}}(\frac{\partial g}{\partial v_{n+1}}{)}^{-1}\frac{\partial g}{\partial u_{n+1}},\\ Q(t) & =\frac{\partial f}{\partial u_{n+1-m}}-\frac{\partial f}{\partial v_{n+1}}(\frac{\partial g}{\partial v_{n+1}}{)}^{-1}\frac{\partial g}{\partial u_{n+1-m}}\\ & -\frac{\partial f}{\partial v_{n+1-m}}(\frac{\partial g}{\partial v_{n+1-m}}{)}^{-1}\frac{\partial g}{\partial u_{n+1-m}},\\ R(t) & =-\frac{\partial f}{\partial v_{n+1-m}}(\frac{\partial g}{\partial v_{n+1-m}}{)}^{-1}\frac{\partial g}{\partial u_{n+1-2m}}. \end{array} \end{array}$$

Simplifying the expression above yields


$$\sigma_{n+1}={\left(I-hH^{T}P(t)H\right)}^{-1}\left(\sigma_{n}+hH^{T}Q(t)H\cdot \sigma_{n+1-m}+hH^{T}R(t)H\sigma_{n+1-2m}\right).$$ (21)

**Theorem 4.1.** *Under the conditions of Theorem 3.1, the implicit Euler method are stable for nonlinear DDAEs with 2-delays.*

**Proof***. Taking norm of* $\sigma_{n}$*, and letting $Y_{n}=\|\sigma_{n}\|,\;0\leq n\leq m-1$, from (20), (21), and condition (10), we get*


$$\begin{array}{c} \begin{array}{cc} & \;Y_{n+1}\\ & =\left\|{\left(I-hH^{T}P(t)H\right)}^{-1}\cdot \left(\sigma_{n}+hH^{T}Q(t)H\cdot \sigma_{n+1-m}+hH^{T}R(t)H\cdot \sigma_{n+1-2m}\right)\right\|\\ & \leq \left\|{\left(I-hH^{T}P(t)H\right)}^{-1}\right\|\cdot \left(Y_{n}+h\|H^{T}Q(t)H\|Y_{n+1-m}+h\|H^{T}R(t)H\|Y_{n+1-2m}\right)\\ & \leq \frac{1+h\gamma (t)+h\varepsilon (t)}{1-h\lambda_{1}(t)}\max_{-2\tau \leq t\leq 0}\|H^{T}\|\cdot \|\Phi (t)-\tilde{\Phi }(t)\|\\ & \leq \max_{-2\tau \leq t\leq 0}\|H^{T}\|\cdot \|\Phi (t)-\tilde{\Phi }(t)\|. \end{array} \end{array}$$ (22)

*It is easy to verify for* $\left\|u(t_{n})-\tilde{u}(t_{n})\right\|$ *and* $\left\|v(t_{n})-\tilde{v}(t_{n})\right\|$ *that*


$$\|u_{n}-{\tilde{u}}_{n}\|\leq \max_{-2\tau \leq t\leq 0}\|\Phi (t)-\tilde{\Phi }(t)\|,$$

*and*


$$\|v_{n}-{\tilde{v}}_{n}\|\leq L\max_{-2\tau \leq t\leq 0}\|\Phi (t)-\tilde{\Phi }(t)\|,$$

*where* $L=\max_{0\leq t\leq 2\tau }\left(\left\|(\frac{\partial g}{\partial v_{n}}{)}^{-1}\frac{\partial g}{\partial u_{n}}\right\|+\left\|(\frac{\partial g}{\partial v_{n}}{)}^{-1}\frac{\partial g}{\partial u_{n-m}}\right\|\right)$.

**Theorem 4.2.** *Under the conditions stated above Theorem 3.1, if systems of* (18)-(19) *satisfy*


$$\gamma (t)+\varepsilon (t)\leq -\xi \lambda_{1}(t),\;0\leq \xi <1,\;-\lambda_{1}(t)<\beta ,\;\beta >0.$$ (23)

*the implicit Euler methods are asymptotically stable for nonlinear DDAEs with 2-delays.*

**Proof***. Let* 0 ≤ *n* ≤ *m* − 1*, we get from* (22) *that*


$$Y_{n+1}\leq \frac{1+h\gamma (t)+h\varepsilon (t)}{1-h\lambda_{1}(t)}\cdot \max_{-2\tau \leq t\leq 0}\|H^{T}\|\cdot \|\Phi (t)-\tilde{\Phi }(t)\|.$$

*From condition (23) and the coefficients in the expression above yield*


$$\frac{1+h\gamma (t)+h\varepsilon (t)}{1-h\lambda_{1}(t)}\leq \frac{1-h\xi \lambda_{1}(t)}{1-h\lambda_{1}(t)}=\frac{1+h\xi \beta }{1+h\beta }=:\eta <1.$$ (24)

*Therefore, when* 0 ≤ *n* ≤ *m* − 1*, we obtain from the above inequality that*


$$Y_{n+1}\leq \eta \max_{-2\tau \leq t\leq 0}\|H^{T}\|\cdot \|\Phi (t)-\tilde{\Phi }(t)\|.$$

*For the case of m = n, we get from (22) and condition (23) that*


$$\begin{array}{c} \begin{array}{cc} Y_{n+1} & \leq \frac{Y_{n}+h(\gamma (t)\cdot Y_{1}+\varepsilon (t)\cdot Y_{1-n})}{1-h\lambda_{1}(t)}\\ & \leq \frac{Y_{n}+h(\gamma (t)+\varepsilon (t))Y_{1}}{1-h\lambda_{1}(t)}\cdot \max_{-2\tau \leq t\leq 0}\|H^{T}||\cdot \|\Phi (t)-\tilde{\Phi }(t)\|\\ & \leq \eta^{2}\cdot \max_{-2\tau \leq t\leq 0}\|H^{T}||\cdot \|\Phi (t)-\tilde{\Phi }(t)\|. \end{array} \end{array}$$

*For the case* *rm* ≤ *n* ≤ (*r* + 1)*m* − 1 *with* r=1,2,⋯*, it is shown by induction that*


$$Y_{n+1}\leq \eta^{r}\max_{-2\tau \leq t\leq 0}\|H^{T}||\cdot \|\Phi (t)-\tilde{\Phi }(t)\|.$$

*Using the inequality above and* $\left\|{\bar{Y}}_{n+1}\|=\|H^{T}\|\cdot \|u_{n+1}-{\tilde{u}}_{n+1}\|\;(\|H^{T}\|\neq 0\right)$*, we obtain*


$$\begin{array}{c} \begin{array}{cc} \left\|u_{n+1}-{\tilde{u}}_{n+1}\right\| & \leq \eta^{r+1}\max_{-2\tau \leq t\leq 0}\|H^{T}||\cdot \|\Phi (t)-\tilde{\Phi }(t)\|,\\ \left\|v_{n+1}-{\tilde{v}}_{n+1}\right\| & \leq \eta^{r+1}L\max_{-2\tau \leq t\leq 0}\|H^{T}||\cdot \|\Phi (t)-\tilde{\Phi }(t)\|. \end{array} \end{array}$$

*As* r→∞ *and* n→∞*, condition (24) implies that*


$$\lim_{n\to \infty }\|u_{n}-{\tilde{u}}_{n}\|\to 0,\;\lim_{n\to \infty }\|v_{n}-{\tilde{v}}_{n}\|\to 0.$$

### 4.2 The 2-step BDF method

Applying the 2-step BDF method to (1) and linearizing directly in a neighborhood of the stationary solution yields


$$\begin{array}{c} \left\{ \begin{array}{cc} u_{n+2} & =\frac{4}{3}u_{n+1}-\frac{1}{3}u_{n}+\frac{2}{3}h(\frac{\partial f}{\partial u_{n+2}}u_{n+2}+\frac{\partial f}{\partial u_{n+2-m}}u_{n+2-m})\\ & +\frac{2}{3}h(\frac{\partial f}{\partial v_{n+2}}v_{n+2}+\frac{\partial f}{\partial_{n+2-m}}v_{n+2-m}),\;n=1,2,\cdots ,\\ 0 & =\frac{\partial g}{\partial u_{n+1}}u_{n+1}+\frac{\partial g}{\partial u_{n+1-m}}u_{n+1-m}+\frac{\partial g}{\partial v_{n+1}}v_{n+1},\\ u_{n} & =\Phi_{1}(t_{n}),\;v_{n}=\Psi (t_{n}),\;-m\leq n\leq 0,\;(mh=\tau ,\;m\geq 1), \end{array}\right. \end{array}$$ (25)

where


$$\begin{array}{c} u_{n}=\Phi (t_{n})=\left\{ \begin{array}{cc} \phi_{1}(t_{n}),\; & t_{n}\in [-\tau ,0],\\ \phi_{2}(t_{n}),\; & t_{n}\in [-2\tau ,\tau ), \end{array}\right. \end{array}$$

$$\begin{array}{c} v_{n}=\Psi (t_{n})=\left\{ \begin{array}{cc} \psi_{1}(t_{n}),\; & t_{n}\in [-\tau ,0],\\ \psi_{2}(t_{n}),\; & t_{n}\in [-2\tau ,\tau ), \end{array}\right. \end{array}$$

By considering only a local perturbation of (25) to examine small perturbation trends and neglecting higher-order terms, we obtain


$$\begin{array}{c} \left\{ \begin{array}{cc} {\tilde{u}}_{n+2} & =\frac{4}{3}{\tilde{u}}_{n+1}-\frac{1}{3}{\tilde{u}}_{n}+\frac{2}{3}h(\frac{\partial f}{\partial u_{n+2}}{\tilde{u}}_{n+2}+\frac{\partial f}{\partial u_{n+2-m}}{\tilde{u}}_{n+2-m})\\ & +\frac{2}{3}h(\frac{\partial f}{\partial v_{n+2}}{\tilde{v}}_{n+2}+\frac{\partial f}{\partial_{n+2-m}}{\tilde{v}}_{n+2-m}),\;n=1,2,\cdots ,\\ 0 & =\frac{\partial g}{\partial u_{n+1}}{\tilde{u}}_{n+1}+\frac{\partial g}{\partial u_{n+1-m}}{\tilde{u}}_{n+1-m}+\frac{\partial g}{\partial v_{n+1}}{\tilde{v}}_{n+1},\\ {\tilde{u}}_{n} & =\tilde{\Phi }(t_{n}),\;{\tilde{v}}_{n}=\tilde{\Psi }(t_{n}),\;-m\leq n\leq 0,\;(mh=\tau ,\;m\geq 1), \end{array}\right. \end{array}$$ (26)

where


$$\begin{array}{c} {\tilde{u}}_{n}=\tilde{\Phi }(t_{n})=\left\{ \begin{array}{cc} {\tilde{\phi }}_{1}(t_{n}),\; & t_{n}\in [-\tau ,0],\\ {\tilde{\phi }}_{2}(t_{n}),\; & t_{n}\in [-2\tau ,\tau ), \end{array}\right. \end{array}$$

$$\begin{array}{c} {\tilde{v}}_{n}=\tilde{\Psi }(t_{n})=\left\{ \begin{array}{cc} {\tilde{\psi }}_{1}(t_{n}),\; & t_{n}\in [-\tau ,0],\\ {\tilde{\psi }}_{2}(t_{n}),\; & t_{n}\in [-2\tau ,\tau ). \end{array}\right. \end{array}$$

Following similar approach as stability analysis in 3.1, there exists an orthogonal matrix *H* satisfies (5). Given that *H*^*T*^*P*(*t*)*H* is a negative definite diagonal matrix, we immediately obtain (20). From systems (25) and (26), we get the similar conclusion as (7)-(9). Letting $\sigma_{n}=H^{T}(u_{n}-{\tilde{u}}_{n})$, and proceeding analogously to (11), we obtain


$$\begin{array}{c} \begin{array}{cc} \sigma_{n+2} & =\sigma_{n+1}+\frac{1}{3}(\sigma_{n+1}-\sigma_{n})+\frac{2}{3}h\cdot H^{T}P(t)H\cdot \sigma_{n+2}\\ & +\frac{2}{3}h\cdot \left(H^{T}Q(t)H\cdot \sigma_{n+2-m}+H^{T}R(t)H\cdot \sigma_{n+2-2m}\right), \end{array} \end{array}$$ (27)

where


$$\begin{array}{c} \begin{array}{cc} P(t) & =\frac{\partial f}{\partial u_{n+2}}-\frac{\partial f}{\partial v_{n+2}}(\frac{\partial g}{\partial v_{n+2}}{)}^{-1}\frac{\partial g}{\partial u_{n+2}},\\ \left[10pt]Q(t\right) & =\frac{\partial f}{\partial u_{n+1-m}}-\frac{\partial f}{\partial v_{n+1}}(\frac{\partial g}{\partial v_{n+1}}{)}^{-1}\frac{\partial g}{\partial u_{n+1-m}}\\ \left[10pt\right] & -\frac{\partial f}{\partial v_{n+1-m}}(\frac{\partial g}{\partial v_{n+1-m}}{)}^{-1}\frac{\partial g}{\partial u_{n+1-m}},\\ \left[10pt]R(t\right) & =-\frac{\partial f}{\partial v_{n+1-m}}(\frac{\partial g}{\partial v_{n+1-m}}{)}^{-1}\frac{\partial g}{\partial u_{n+1-2m}}. \end{array} \end{array}$$

Simplifying the above expression gives


$$\begin{array}{c} \begin{array}{cc} \sigma_{n+2} & ={\left(I-\frac{2}{3}hH^{T}P(t)H\right)}^{-1}\\ & \cdot \left\{\sigma_{n+1}+\frac{1}{3}(\sigma_{n+1}-\sigma_{n})+\frac{2}{3}h(H^{T}Q(t)H\cdot \sigma_{n+2-m}+H^{T}R(t)H\cdot \sigma_{n+2-2m})\right\}. \end{array} \end{array}$$ (28)

**Theorem 4.3.** *Under the conditions of Theorem 3.1, if*


$$\begin{array}{c} \frac{1}{2}\omega (t)+\gamma (t)+\varepsilon (t)\leq -\lambda_{1}(t),\;\omega (t)=-\lambda_{1}(t)+\gamma (t)+\varepsilon (t). \end{array}$$ (29)

*then the 2-step BDF method is stable for nonlinear DDAEs with 2-delays.*

**Proof***. We now proceed analogously to the proof of Theorem 4.1. Taking the norm of* $\sigma_{n}$ *for* 0 ≤ *n* ≤ *m* − 1*, and letting* $Y_{n}=\|\sigma_{n}\|$*, we obtain from* (20), (28)*, and condition* (10) *that*


$$\begin{array}{c} \begin{array}{cc} Y_{n+2} & =\|(I-\frac{2}{3}hH^{T}P(t)H{)}^{-1}\cdot (\sigma_{n+1}+\frac{1}{3}(\sigma_{n+1}-\sigma_{n})\\ & +\frac{2}{3}h(H^{T}Q(t)H\cdot \sigma_{n+2-m}+H^{T}R(t)H\cdot \sigma_{n+2-2m}))\|\\ & \leq \|(I-\frac{2}{3}hH^{T}P(t)H{)}^{-1}\|\cdot (Y_{n+1}+\frac{1}{3}\|\sigma_{n+1}-\sigma_{n}\|\\ & +\frac{2}{3}h(\|H^{T}Q(t)H\|\cdot Y_{n+2-m}+\|H^{T}R(t)H\|\cdot Y_{n+2-2m}))\\ & \leq \frac{Y_{n+1}+\frac{1}{3}\|\sigma_{n+1}-\sigma_{n}\|+\frac{2}{3}h(\gamma \cdot Y_{n+2-m}+\varepsilon \cdot Y_{n+2-2m})}{I-\frac{2}{3}h\lambda_{1}(t)} \end{array} \end{array}$$ (30)

*With the initial values u*_*0*_*, u*_*1*_, ${\tilde{u}}_{0}$, ${\tilde{u}}_{1}$ *for the 2-step BDF method, we have*


$$\begin{array}{c} \begin{array}{cc} \left\|Y_{0}\right\| & =\|H^{T}\cdot (u_{0}-{\tilde{u}}_{0})\|\leq \max_{-2\tau \leq t\leq 0}\|H^{T}\|\cdot \|\Phi (t)-\tilde{\Phi }(t)\|,\\ \left\|Y_{1}\right\| & =\|H^{T}\cdot (u_{1}-{\tilde{u}}_{1})\|\leq \max_{-2\tau \leq t\leq 0}\|H^{T}\|\cdot \|\Phi (t)-\tilde{\Phi }(t)\|. \end{array} \end{array}$$ (31)

*For the case n = 0, the implicit Euler method can be used to compute* $\sigma_{1}-\sigma_{0}$ *as follows*


$$\begin{array}{c} \begin{array}{cc} \sigma_{1}-\sigma_{0} & =h\sigma_{1}^{\prime }=h\left(H^{T}P(t)H\cdot \sigma_{1}+H^{T}Q(t)H\cdot \sigma_{1-m}+H^{T}R(t)H\cdot \sigma_{1-2m}\right),\\ \left\|\sigma_{1}-\sigma_{0}\right\| & =hY_{1}^{\prime }\leq h\left(-\lambda_{1}(t)\cdot Y_{1}+\gamma (t)\cdot Y_{1-m}+\varepsilon (t)\cdot Y_{1-2m}\right)\\ & \leq h\left(-\lambda_{1}(t)+\gamma (t)+\varepsilon (t)\right)\max_{-2\tau \leq t\leq 0}\|H^{T}\|\cdot \|\Phi (t)-\tilde{\Phi }(t)\|\\ & =h\omega (t)\max_{-2\tau \leq t\leq 0}\|H^{T}\|\cdot \|\Phi (t)-\tilde{\Phi }(t)\|. \end{array} \end{array}$$

*Substituting the above expression into (30) and applying condition (29), we obtain*


$$\begin{array}{c} \begin{array}{cc} Y_{2} & \leq \frac{Y_{1}+\frac{1}{3}\|\sigma_{1}-\sigma_{0}\|+\frac{2}{3}h\cdot \left(\gamma (t)\cdot Y_{2-m}+\varepsilon (t)\cdot Y_{2-2m}\right)}{1-\frac{2}{3}h\lambda_{1}(t)}\\ & \leq \frac{1+\frac{1}{3}h\omega (t)+\frac{2}{3}h\cdot (\gamma (t)+\varepsilon (t))}{1-\frac{2}{3}h\lambda_{1}(t)}\cdot \max_{-2\tau \leq t\leq 0}\|H^{T}\|\cdot \|\Phi (t)-\tilde{\Phi }(t)\|\\ & \leq \max_{-2\tau \leq t\leq 0}\|H^{T}\|\cdot \|\Phi (t)-\tilde{\Phi }(t)\|. \end{array} \end{array}$$ (32)

*For the case n = 1, the 2-step BDF method can be used to compute* $\sigma_{2}-\sigma_{1}$*, and taking its norm yields*


$$\begin{array}{c} \begin{array}{cc} \sigma_{2}-\sigma_{1} & =\frac{1}{3}(\sigma_{1}-\sigma_{0})+\frac{2}{3}h\sigma_{2}^{\prime },\\ \left\|\sigma_{2}-\sigma_{1}\right\| & \leq \frac{1}{3}\|\sigma_{1}-\sigma_{0}\|+\frac{2}{3}h\left(-\lambda_{1}(t)\cdot Y_{2}+\gamma (t)\cdot Y_{2-m}+\varepsilon (t)\cdot Y_{2-2m}\right)\\ & \leq \left(\frac{1}{3}h\omega (t)+\frac{2}{3}h\left(-\lambda_{1}(t)+\gamma (t)+\varepsilon (t)\right)\right)\cdot \max_{-2\tau \leq t\leq 0}\|H^{T}\|\cdot \|\Phi (t)-\tilde{\Phi }(t)\|\\ & =h\omega (t)\cdot \max_{-2\tau \leq t\leq 0}\|H^{T}\|\cdot \|\Phi (t)-\tilde{\Phi }(t)\|. \end{array} \end{array}$$

*Substituting the above expression into (30) and employing condition (29) and together with inequality (32), we obtain*


$$\begin{array}{c} \begin{array}{cc} Y_{3} & \leq \frac{Y_{2}+\frac{1}{3}\|\sigma_{2}-\sigma_{1}\|+\frac{2}{3}h\left(\gamma (t)\cdot Y_{3-m}+\varepsilon (t)\cdot Y_{3-2m}\right)}{1-\frac{2}{3}h\lambda_{1}(t)}\\ & \leq \frac{1+\frac{1}{3}h\omega (t)+\frac{2}{3}h\cdot \left(\gamma (t)+\varepsilon (t)\right)}{1-\frac{2}{3}h\lambda_{1}(t)}\cdot \max_{-2\tau \leq t\leq 0}\|H^{T}\|\cdot \|\Phi (t)-\tilde{\Phi }(t)\|\\ & \leq \max_{-2\tau \leq t\leq 0}\|H^{T}\|\cdot \|\Phi (t)-\tilde{\Phi }(t)\|. \end{array} \end{array}$$ (33)

*For the case n = 2, the 2-step BDF method can be applied to compute* $\sigma_{3}-\sigma_{2}$*, and taking its norm leads to*


$$\begin{array}{c} \begin{array}{cc} \sigma_{3}-\sigma_{2} & =\frac{1}{3}(\sigma_{2}-\sigma_{1})+\frac{2}{3}h\sigma_{3}^{\prime }=\frac{1}{3}\left(\frac{1}{3}(\sigma_{1}-\sigma_{0})+\frac{2}{3}h\sigma_{2}^{\prime }\right)+\frac{2}{3}h\sigma_{3}^{\prime }\\ & =(\frac{1}{3}{)}^{2}(\sigma_{1}-\sigma_{0})+\frac{1}{3}\cdot \frac{2}{3}h\sigma_{2}^{\prime }+\frac{2}{3}h\sigma_{3}^{\prime },\\ \left\|\sigma_{3}-\sigma_{2}\right\| & \leq (\frac{1}{3}{)}^{2}\|\sigma_{1}-\sigma_{0}\|+\frac{1}{3}\cdot \frac{2}{3}h\|\sigma_{2}^{\prime }\|+\frac{2}{3}h\|\sigma_{3}^{\prime }\|\\ & \leq (\frac{1}{3}{)}^{2}\|\sigma_{2}-\sigma_{1}\|+\frac{1}{3}\cdot \frac{2}{3}\cdot h\left(-\lambda_{1}(t)Y_{2}+\gamma (t)Y_{2-m}+\varepsilon (t)Y_{2-2m}\right)\\ & +\frac{2}{3}\cdot h\left(-\lambda_{1}(t)Y_{3}+\gamma (t)Y_{3-m}+\varepsilon (t)Y_{3-2m}\right)\\ & \leq h\left((\frac{1}{3}{)}^{2}+\frac{1}{3}\cdot \frac{2}{3}+\frac{2}{3}\right)\cdot \omega (t)\max_{-2\tau \leq t\leq 0}\|H^{T}\|\cdot \|\Phi (t)-\tilde{\Phi }(t)\|\\ & =h\omega (t)\max_{-2\tau \leq t\leq 0}\|H^{T}\|\cdot \|\Phi (t)-\tilde{\Phi }(t)\|. \end{array} \end{array}$$

*Substituting the above expression into* equation (30) *and applying condition (29) along with inequalities (32) and (33), we obtain*


$$\begin{array}{c} \begin{array}{cc} Y_{4} & \leq \frac{Y_{3}+\frac{1}{3}\|\sigma_{3}-\sigma_{2}\|+\frac{2}{3}h(\gamma (t)\cdot Y_{4-m}+\varepsilon (t)\cdot Y_{4-2m})}{1-\frac{2}{3}h\lambda_{1}(t)}\\ & \leq \frac{1+\frac{1}{3}h\omega (t)+\frac{2}{3}h\left(\gamma (t)+\varepsilon (t)\right)}{1-\frac{2}{3}h\lambda_{1}(t)}\cdot \max_{-2\tau \leq t\leq 0}\|H^{T}\|\cdot \|\Phi (t)-\tilde{\Phi }(t)\|\\ & \leq \max_{-2\tau \leq t\leq 0}\|H^{T}\|\cdot \|\Phi (t)-\tilde{\Phi }(t)\|. \end{array} \end{array}$$ (34)

*Since*


$$(\frac{1}{3}{)}^{n}+\frac{2}{3}h\sum_{i=2}^{n+1}(\frac{1}{3}{)}^{n+1-i}=1,\;n=0,1,2,\cdots ,$$

*for the cases* n=3,4,…*, the 2-step BDF method can be used to compute* $\sigma_{n+1}-\sigma_{n}$*, and taking norms of* $\sigma_{n+1}-\sigma_{n}$ *separately. Then, by mathematical induction, we obtain*


$$\begin{array}{c} \begin{array}{cc} \sigma_{n+1}-\sigma_{n} & =(\frac{1}{3}{)}^{n}(\sigma_{1}-\sigma_{0})+(\frac{1}{3}{)}^{n-1}\cdot \frac{2}{3}h\sigma_{2}^{\prime }+(\frac{1}{3}{)}^{n-2}\cdot \frac{2}{3}h\sigma_{3}^{\prime }+\cdots +\frac{2}{3}h\sigma_{n+1}^{\prime }\\ \left[3pt\right] & =(\frac{1}{3}{)}^{n}(\sigma_{1}-\sigma_{0})+\frac{2}{3}h\sum_{i=2}^{n+1}(\frac{1}{3}{)}^{n+1-i}\sigma_{i}^{\prime }.\\ \left[3pt]\|\sigma_{n+1}-\sigma_{n}\right\| & \leq (\frac{1}{3}{)}^{n}\|\sigma_{1}-\sigma_{0}\|+\frac{2}{3}h\sum_{i=2}^{n+1}(\frac{1}{3}{)}^{n+1-i}\|\sigma_{i}^{\prime }\|\\ \left[3pt\right] & \leq (\frac{1}{3}{)}^{n}\|\sigma_{1}-\sigma_{0}\|+\frac{2}{3}h\sum_{i=2}^{n+1}(\frac{1}{3}{)}^{n+1-i}\cdot \left(-\lambda_{1}(t)Y_{i}+\gamma (t)Y_{i-m}+\varepsilon (t)Y_{i-2m}\right)\\ \left[3pt\right] & \leq h\left((\frac{1}{3}{)}^{n}+\frac{2}{3}\sum_{i=2}^{n+1}(\frac{1}{3}{)}^{n+1-i}\right)\omega (t)\max_{-2\tau \leq t\leq 0}\|H^{T}\|\cdot \|\Phi (t)-\tilde{\Phi }(t)\|\\ \left[3pt\right] & =h\omega (t)\cdot \max_{-2\tau \leq t\leq 0}\|H^{T}\|\cdot \|\Phi (t)-\tilde{\Phi }(t)\|. \end{array} \end{array}$$

*Substituting the above expression into* equation (30) *and employing condition (29) together with inequalities (32), (33), and (34), we obtain*


$$\begin{array}{c} \begin{array}{cc} Y_{n+2} & \leq \frac{Y_{n+1}+\frac{1}{3}\|\sigma_{n+1}-\sigma_{n}\|+\frac{2}{3}h\left(\gamma (t)\cdot Y_{n+2-m}+\varepsilon (t)\cdot Y_{n+2-2m}\right)}{1-\frac{2}{3}h\lambda_{1}(t)}\\ & \leq \frac{1+\frac{1}{3}h\cdot \omega (t)+\frac{2}{3}h\left(\gamma (t)+\varepsilon (t)\right)}{1-\frac{2}{3}h\lambda_{1}(t)}\cdot \max_{-2\tau \leq t\leq 0}\|H^{T}\|\cdot \|\Phi (t)-\tilde{\Phi }(t)\|\\ & \leq \max_{-2\tau \leq t\leq 0}\|H^{T}\|\cdot \|\Phi (t)-\tilde{\Phi }(t)\|. \end{array} \end{array}$$ (35)

*According to the relation* $Y_{n}=\|H^{T}\|\cdot \|u_{n}-{\tilde{u}}_{n}\|$*, it is easy to observe that*


$$\|u_{n}-{\tilde{u}}_{n}\|\leq \max_{-2\tau \leq t\leq 0}\|\Phi (t)-\tilde{\Phi }(t)\|,\;\|v_{n}-{\tilde{v}}_{n}\|\leq L\cdot \max_{-2\tau \leq t\leq 0}\|\Phi (t)-\tilde{\Phi }(t)\|.$$

**Theorem 4.4.** *Under the conditions of Theorem 3.1, if*


$$\frac{1}{2}\omega (t)+\gamma (t)+\varepsilon (t)\leq -\xi \lambda_{1}(t),\;0\leq q<1,\;\lambda_{1}(t)<-\beta ,\;\beta >0,$$ (36)

*then the 2-step BDF method is asymptotically stable for DDAEs with 2-delays.*

**Proof.** *Letting* 0 ≤ *n* ≤ *m* − 1*, and from inequality* (35) *to obtain*


$$Y_{n+2}\leq \frac{1+\frac{1}{3}h\omega (t)+\frac{2}{3}h\left(\gamma (t)+\varepsilon (t)\right)}{1-\frac{2}{3}h\lambda_{1}(t)}\cdot \max_{-2\tau \leq t\leq 0}\|H^{T}\|\cdot \|\Phi (t)-\tilde{\Phi }(t)\|.$$

*It is clear from (36) that the coefficient in the above inequality satisfies the condition*


$$\begin{array}{c} \frac{1+\frac{1}{3}h\omega (t)+\frac{2}{3}h(\gamma (t)+\varepsilon (t))}{1-\frac{2}{3}h\lambda_{1}(t)}\leq \frac{1-\frac{2}{3}h\xi \lambda_{1}(t)}{1-\frac{2}{3}h\lambda_{1}(t)}<\frac{1+\frac{2}{3}h\xi \beta }{1+\frac{2}{3}h\beta }<1. \end{array}$$ (37)

*Consequently, for* 0 ≤ *n* ≤ *m* − 1*, by setting* $\eta =\frac{1+\frac{2}{3}h\xi \beta }{1+\frac{2}{3}h\beta }$ *with* 0<η<1*, we obtain the bound*


$$Y_{n}\leq \eta \cdot \max_{-2\tau \leq t\leq 0}\|H^{T}\|\cdot \|\Phi (t)-\tilde{\Phi }(t)\|.$$

*For the case m = n, combining (35) with the result of (37) gives*


$$\begin{array}{c} \begin{array}{cc} Y_{n+2} & \leq \frac{Y_{n+1}+\frac{1}{3}\|\sigma_{n+1}-\sigma_{n}\|+\frac{2}{3}h\left(\gamma (t)\cdot Y_{2}+\varepsilon (t)\cdot Y_{2-m}\right)}{1-\frac{2}{3}h\lambda_{1}(t)}\\ & \leq \frac{1+\frac{1}{3}h\omega (t)+\frac{2}{3}h\left(\gamma (t)+\varepsilon (t)\right)}{1-\frac{2}{3}h\lambda_{1}(t)}\eta \cdot \max_{-2\tau \leq t\leq 0}\|H^{T}\|\cdot \|\Phi (t)-\tilde{\Phi }(t)\|\\ & \leq \eta^{2}\cdot \max_{-2\tau \leq t\leq 0}\|H^{T}\|\cdot \|\Phi (t)-\tilde{\Phi }(t)\|. \end{array} \end{array}$$

*When* *rm* ≤ *n* ≤ (*r* + 1)*m* − 1*, by mathematical induction yields*


$$Y_{n+2}\leq \eta^{r+2}\cdot \max_{-2\tau \leq t\leq 0}\|H^{T}\|\cdot \|\Phi (t)-\tilde{\Phi }(t)\|.$$

*Since* 0<η<1*, it follows that*


$$\lim_{r\to \infty }Y_{n+2}\to 0.$$

*Recalling that* $Y_{n}=\|H^{T}\|\cdot \|u_{n}-{\tilde{u}}_{n}\|$ *and noting that* *L* < ∞*, we obtain*


$$\begin{array}{c} \begin{array}{cc} \left\|u_{n}-{\tilde{u}}_{n}\right\| & \leq \eta^{r}\cdot \max_{-2\tau \leq t\leq 0}\|\Phi (t)-\tilde{\Phi }(t)\|,\\ \left\|v_{n}-{\tilde{v}}_{n}\right\| & \leq L\|u_{n}-{\tilde{u}}_{n}\|\leq L\eta^{r}\cdot \max_{-2\tau \leq t\leq 0}\|\Phi (t)-\tilde{\Phi }(t)\|. \end{array} \end{array}$$

*Finally, taking the limit as* r→∞*, we conclude*


$$\lim_{n\to \infty }\|u_{n}-{\tilde{u}}_{n}\|\to 0,\;\lim_{n\to \infty }\|v_{n}-{\tilde{v}}_{n}\|\to 0.$$

## 5 Numerical examples

**Example 5.1.** *Let* u(t),v(t)∈R*,* f:R×R×R×R×R→R,g:R×R→R*.*


$$\begin{array}{c} \left\{ \begin{array}{cc} u^{\prime }(t) & =\alpha (t)u(t)+A_{1}(t)f_{1}(u(t-\tau ))+A_{2}(t)f_{2}(v(t))+A_{3}(t)f_{3}(v(t-\tau )),\\ 0 & =g(u,u(t-\tau ),v), \end{array}\right. \end{array}$$ (38)

*where* $\alpha (t),A_{1}(t),A_{2}(t),A_{3}(t)$ *are polynomials of t, and*


$$\begin{array}{c} \begin{array}{cc} & \alpha (t)=-2(1+t{)}^{2},\;A_{1}(t)=t,\;A_{2}(t)=2t^{2},\;A_{3}(t)=1+t,\\ & f_{1}(u_{\tau })=sinu_{\tau },\;f_{2}(v)=log(1+v^{2}),\;f_{3}(v_{\tau })=cosv_{\tau },\\ & g(u,u_{\tau },v)=u+u_{\tau }-arctan(1+v).\\ & u(t)=t+\frac{1}{2},\;v(t)=\tan2t-1,\;-2\tau \leq t\leq 0. \end{array} \end{array}$$

*Using the direct linearization method for (38) to have*


$$\begin{array}{c} \left\{ \begin{array}{cc} u^{\prime }(t) & =\alpha (t)u(t)+A_{1}(t)\frac{\partial f_{1}(u_{\tau })}{\partial u_{\tau }}u(t-\tau )\\ & +A_{2}(t)\frac{\partial f_{2}(v)}{\partial v}v(t)+A_{3}(t)\frac{\partial f_{3}(v_{\tau })}{\partial v_{\tau }}v(t-\tau ),\\ 0 & =\frac{\partial g}{\partial u}u(t)+\frac{\partial g}{\partial u}u(t-\tau )+\frac{\partial g}{\partial v}v(t), \end{array}\right. \end{array}$$

*Taking* τ=1 *and applying Theorem 3.1, we get*


$$\begin{array}{c} \begin{array}{cc} u^{\prime }(t) & =(-2(1+t{)}^{2}+\frac{2t^{2}(2+(\tan2t-1{)}^{2})}{(1+(\tan2t-1{)}^{2})ln10})\cdot u(t)\\ & +t\cos(t-\frac{1}{2})+\frac{2t^{2}(2+(\tan2(t-1)-1{)}^{2})}{(1+(\tan2(t-1)-1{)}^{2})ln10}\\ & -(2+(\tan2(t-1)-1{)}^{2})(1+t)\sin(t-\frac{1}{2}))\cdot u(t-\tau )\\ & +(2+(\tan2(t-1)-1{)}^{2})(1+t)\sin(\tan2(t-1)-1)\cdot u(t-2\tau ). \end{array} \end{array}$$

*Subsequently, for* t∈[0,2]*, we determine the functions* λ(t), γ(t)*, and* ε(t) *such as*


$$\begin{array}{c} \begin{array}{cc} & \lambda_{1}(t)=-2(1+t{)}^{2}+\frac{2t^{2}(2+(\tan2t-1{)}^{2})}{(1+(\tan2t-1{)}^{2})ln10},\\ & \gamma (t)=t\cos(t-\frac{1}{2})+\frac{2t^{2}(2+(\tan2(t-1)-1{)}^{2})}{(1+(\tan2(t-1)-1{)}^{2})ln10}\\ & -(2+(\tan2(t-1)-1{)}^{2})(1+t)\sin(t-\frac{1}{2}),\\ & \varepsilon (t)=(2+(\tan2(t-1)-1{)}^{2})(1+t)\sin(\tan2(t-1)-1). \end{array} \end{array}$$

*It can be shown that there exist bounded constants L*_*1*_*, L*_*2*_*, and L*_*3*_ *such that*


$$|f(t,x,x_{\tau },x_{2\tau })-f(t,\tilde{x},{\tilde{x}}_{\tau },{\tilde{x}}_{2\tau })|\leq L_{1}\|x-\tilde{x}\|+L_{2}\|x_{\tau }-{\tilde{x}}_{\tau }\|+L_{3}\|x_{2\tau }-{\tilde{x}}_{2\tau }\|.$$

**Fig 1:**
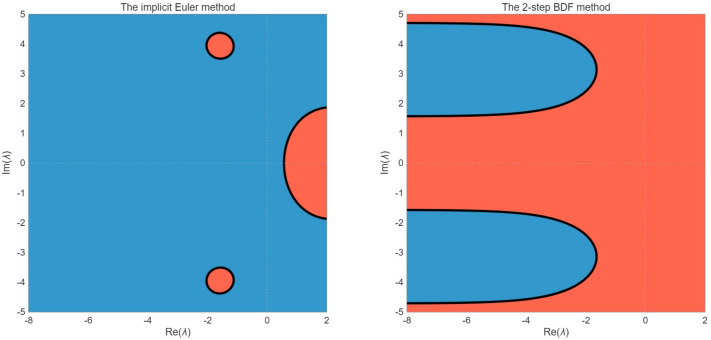
Stability region for the implicit Euler method and the 2-step BDF method for Example 5.1.

*A straightforward verification confirms that all conditions of Theorems 4.1 and 4.3 are satisfied. The stability characteristics of implicit numerical methods for the DDAE with 2-delays system in Example 5.1 are examined through their respective stability regions in the complex* λ*-plane in* Fig 1 *with h = 0.5. For the implicit Euler method, the stability region encompasses nearly the entire left half-plane, demonstrating A-stability with* |R(λ)|<1 *for all* Re(λ)≤0*. This unconditional stability makes it particularly suitable for stiff problems with eigenvalues having large negative real parts, such as the current system where* $\alpha (t)=-2(1+t{)}^{2}$ *produces increasing stiffness over time. In contrast, the 2-step BDF method exhibits a more complex stability region that, while covering substantial portions of the left half-plane, displays instability near the imaginary axis. This conditional stability implies potential limitations when solving oscillatory components of the solution, though the 2-step BDF method offers superior second-order accuracy for non-oscillatory problems. The delay terms in the system, particularly through the expressions* $e^{-\lambda \tau }$ *in the characteristic equations, introduce additional complexity to both stability regions, distorting their boundaries from the classical ODEs case. For the given nonlinear system with multiple delayed terms (*u(t−τ) *and* v(t−τ)*) and algebraic constraints, these linear stability analyses provide essential guidance while noting that complete stability characterization requires consideration of the system’s time-dependent coefficients and nonlinearities.*

**Fig 2:**
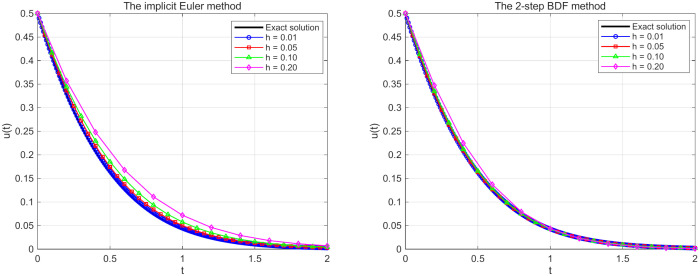
Numerical solutions using numerical methods with different time step sizes for Example 5.1.

Fig 2 *presents the numerical solutions of the delay differential equation using the implicit Euler method and the 2-step BDF method with different time step sizes h = 0.01,0.05,0.10,0.20. Both numerical methods demonstrate convergence behavior that smaller step sizes yield solutions that progressively approach the reference solution. The implicit Euler method has first-order convergence, with noticeable numerical dissipation at larger step sizes, while the 2-step method has second-order accuracy and maintains better solution fidelity across the time domain. These results validate the theoretical convergence properties of the numerical schemes.*

**Fig 3:**
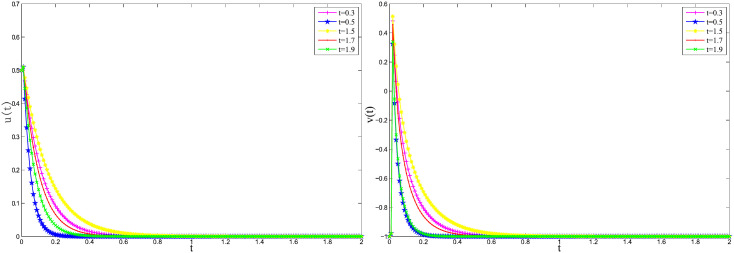
Approximate solutions using the implicit Euler method with *h* = 0.01 for Example 5.1.

**Fig 4:**
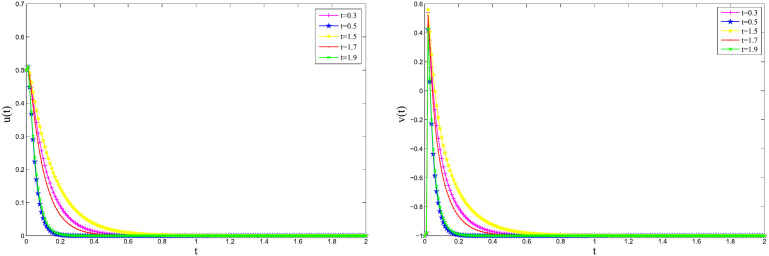
Approximate solutions using the 2-step BDF method with *h* = 0.01 for Example 5.1.


Fig 3 *and* Fig 4 *that the numerical solution curves of the system for different time instants t = 0.3, 0.5, 1.5, 1.7, and 1.9, using the implicit Euler method and the 2-step BDF method, respectively, with step size h = 0.01. The results indicate that under this step size, both numerical methods yield stable solutions. This stability can be attributed to the fact that the initial conditions satisfy the compatibility condition (38). By examining the solution behavior at different time points within the neighborhood of the stationary solution, specifically by varying* $\lambda_{1}(t)$, γ(t) *and* ε(t) *at t = 0.3, 0.5, 1.5, 1.7, and 1.9, it is observed that the numerical solutions remain stable near the steady state. In particular, the system exhibits stability over the intervals 0.3 < t < 0.5 and 1.5 < t < 2. The numerical results further demonstrate that the amplitude of the solution decays over time, eventually approaching zero, which is consistent with the expected asymptotic behavior of the system near its equilibrium.*

**Example 5.2.** *Let* u(t),v(t)∈R*,* f:R×R×R×R×R→R,g:R×R→R*.*

$$\left\{ \begin{array}{c} u^{\prime }(t)=-2u(t)+\frac{1}{e^{2}(1+e^{2})}u(t)v(t-\tau )-\frac{1}{e^{2}(1+e^{2})}u(t-\tau )y(t),\\ 0=\frac{1}{2}u(t)+\frac{1}{2}u(t-\tau )-v(t),\\ u(t)=e^{-2t},\;v(t)=\frac{1}{2}(e^{-2t}+e^{-2t+2}),\;-2\tau \leq t<0. \end{array}\right.$$ (39)

*Taking* τ=1 *and applying Theorem 3.1, we get*


$$\begin{array}{c} \begin{array}{cc} & u^{\prime }(t)=P(t)\cdot u(t)+Q(t)\cdot u(t-\tau )+R(t)\cdot u(t-2\tau ),\\ & P(t)=-2+\frac{1}{2e^{2}(1+e^{2})}(e^{-2(t-1)}+e^{-2(t-1)+2})-\frac{1}{2e^{2}(1+e^{2})}e^{-2(t-1)},\\ & Q(t)=-\frac{1}{2e^{2}(1+e^{2})}(e^{-2t}+e^{-2t+2})-\frac{1}{2e^{2}(1+e^{2})}e^{-2(t-1)}+\frac{1}{2e^{2}(1+e^{2})}e^{-2t},\\ & R(t)=\frac{1}{2e^{2}(1+e^{2})}e^{-2t}. \end{array} \end{array}$$

*Subsequently, for* t∈[0,2]*, we determine the functions* $\lambda_{1}(t)$, γ(t)*, and* ε(t) *such as*


$$\begin{array}{c} \begin{array}{cc} & \lambda_{1}(t)=-2+\frac{1}{2e^{2}(1+e^{2})}e^{-2(t-1)+2},\\ & \gamma (t)=\frac{1}{e^{2}(1+e^{2})}e^{-2(t-1)},\;\varepsilon (t)=\frac{1}{2e^{2}(1+e^{2})}e^{-2t},\\ & \omega (t)=2-\frac{1}{2e^{2}(1+e^{2})}e^{-2(t-1)+2}+\frac{1}{e^{2}(1+e^{2})}e^{-2(t-1)}+\frac{1}{2e^{2}(1+e^{2})}e^{-2t}. \end{array} \end{array}$$

*It can be readily verified that the assumptions of Theorems 4.1 and 4.3 are all satisfied.*

**Fig 5:**
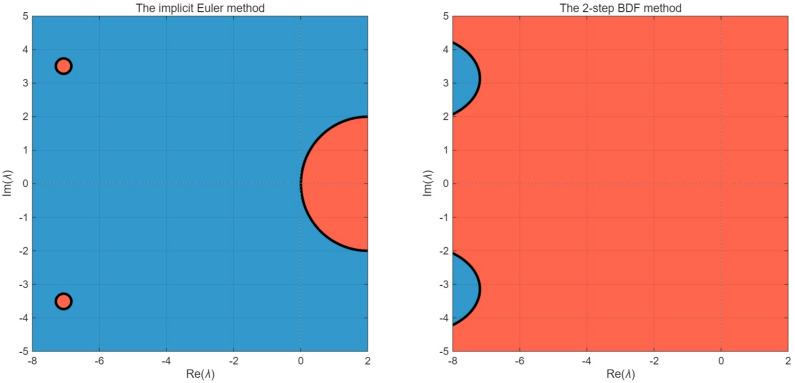
Stability region of the implicit Euler method and the 2-step BDF method for Example 5.2.

*The stability characteristics of implicit numerical methods for the DDAEs system in Example 5.2 is examined through their respective stability regions in the complex* λ*-plane* Fig 5 *illustrates the stability region analysis of the implicit Euler method and the 2-step BDF method for (39) with h = 0.5. Both methods exhibit A-stability, for this DDAEs system, and both methods effectively ensure numerical stability for eigenvalues with negative real parts.*

**Fig 6:**
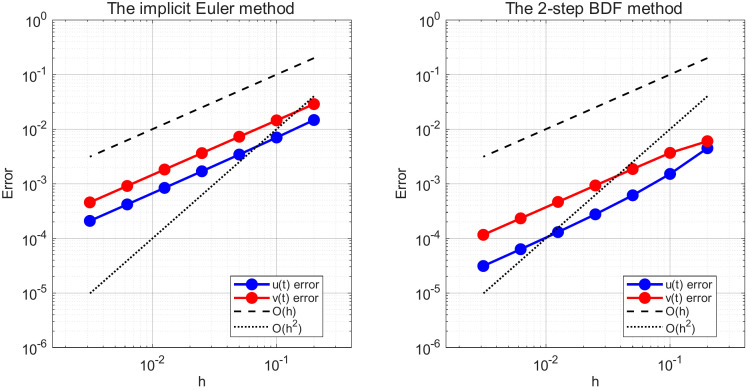
Convergence analysis of the implicit Euler method and the 2-step BDF method for Example 5.2.

*In* Fig 6*, the error of the implicit Euler method decreases at approximately* 𝒪(h)*, reflecting its first-order convergence property, whereas the error of the 2-step BDF method decays at about* $𝒪(h^{2})$*, confirming its second-order convergence rate.*

*Through the comparison of maximum absolute errors between the implicit Euler method and the 2-step BDF method under various step sizes in* Table 1*, we can conduct analysis of the convergence characteristics and computational efficiency of these two numerical approaches. Specifically, when the step size is reduced from 0.1 to* 10^−5^*, the error of the implicit Euler method decreases from* *e*^−2^ *to* *e*^−6^*, a reduction of approximately four orders of magnitude, while under the same conditions, the error of the 2-step BDF method decreases from* *e*^−3^ *to* *e*^−11^*, a reduction of about eight orders of magnitude, demonstrating a significant convergence advantage. Moreover, at a moderate step size h = 0.01, the 2-step BDF method already achieves accuracy comparable to that of the implicit Euler method at an extremely small step size* *h* = 10^−5^*, highlighting the superiority of higher order methods in computational efficiency. The error data also reveal that the numerical error of the algebraic variable v(t) is approximately half that of the differential variable x(t), which aligns with the algebraic constraint* $v(t)=\frac{1}{2}\left(u(t)+u(t-\tau )\right)$ *in the original equation, thereby validating the accuracy of the numerical methods in satisfying algebraic constraints. These results indicate that for this DDAEs system, the 2-step BDF method demonstrates clear advantages over the implicit Euler method in terms of balancing accuracy and efficiency, particularly in scenarios requiring high precision computation.*

**Table 1:** Approximate solutions by using the implicit Euler and the 2-step BDF method for Example 5.2.

		The implicit Euler method		The 2-step BDF method	
*m*	*h*(*mh* = 1)	max$\left|{\bar{u}}_{n}-u_{n}\right|$	max$\left|{\bar{v}}_{n}-v_{n}\right|$	max$\left|{\tilde{u}}_{n}-u_{n}\right|$	max$\left|{\tilde{v}}_{n}-v_{n}\right|$
10	0.1	2.84e-02	1.42e-02	4.20e-03	2.10e-03
20	0.05	1.63e-02	8.10e-03	1.10e-03	5.67e-04
100	0.01	3.60e-03	1.80e-03	4.83e-05	2.42e-05
200	0.005	1.80e-03	9.22e-04	1.22e-05	6.09e-06
1000	0.001	3.73e-04	1.86e-04	4.90e-07	2.45e-07
2000	0.0005	1.87e-04	9.33e-05	1.23e-04	6.13e-08
10^4^	10^−4^	3.74e-05	1.87e-05	4.90e-09	2.45e-09
10^5^	10^−5^	3.74e-06	1.87e-06	4.83e-11	2.41e-11

*u*_*n*_ and *v*_*n*_ are exact solutions, ${\bar{u}}_{n}$ and ${\bar{v}}_{n}$, ${\tilde{u}}_{n}$ and ${\tilde{v}}_{n}$ respectively express numerical solutions by the implicit Euler method and the 2-step BDF method.

**Fig 7:**
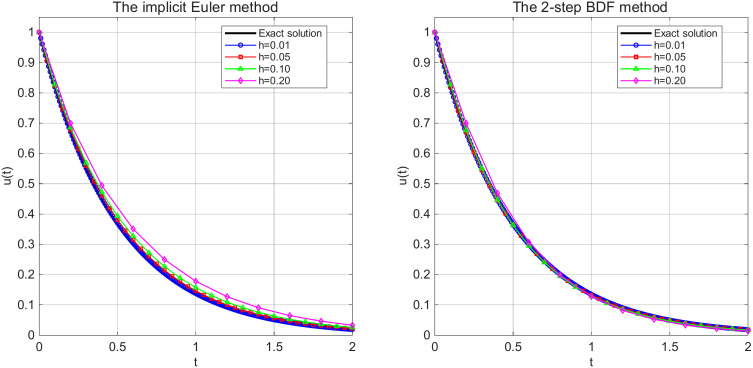
Numerical solutions using numerical methods with different time step sizes for Example 5.2.

Fig 7 *demonstrates that as the step size h decreases, the numerical solutions from both methods progressively approach the exact solution. The implicit Euler method exhibits noticeable numerical dissipation at larger step sizes h = 0.20, producing a smoother solution curve, as the step size reduces to h = 0.01, the numerical solution essentially coincides with the exact solution. The 2-step BDF method shows higher accuracy at equivalent step sizes, achieving satisfactory approximation to the exact solution even at h = 0.05. Overall, both methods demonstrate numerical stability and convergence, for the 2-step BDF method, with its second-order accuracy, can employ larger step sizes while maintaining comparable accuracy, resulting in higher computational efficiency.*

## 6 Results and discussion

This paper presents a stability analysis framework based on direct linearization for the numerical solution of nonlinear DDAEs with 2-delays. The study demonstrates that the implicit Euler and the 2-step BDF method can preserve stability properties of the original systems under appropriate conditions. Through theoretical analysis and numerical verification, the sufficient conditions required for stability were established, thereby enabling the maintenance of stability and asymptotic stability characteristics during the transition from a continuous system to a discrete system. Numerical results indicate that the implicit Euler method has a good ability to maintain stability, while the 2-step BDF method maintains stability with higher computational efficiency. This study provides theoretical foundations for the numerical simulation of delay systems, and can help guide the selection of reliable simulation tools in applications such as networked control systems, biological modeling and power system analysis.
